# SNPrune: an efficient algorithm to prune large SNP array and sequence datasets based on high linkage disequilibrium

**DOI:** 10.1186/s12711-018-0404-z

**Published:** 2018-06-26

**Authors:** Mario P. L. Calus, Jérémie Vandenplas

**Affiliations:** Animal Breeding and Genomics, Wageningen University & Research, P.O. Box 338, 6700 AH Wageningen, The Netherlands

## Abstract

**Background:**

High levels of pairwise linkage disequilibrium (LD) in single nucleotide polymorphism (SNP) array or whole-genome sequence data may affect both performance and efficiency of genomic prediction models. Thus, this warrants pruning of genotyping data for high LD. We developed an algorithm, named SNPrune, which enables the rapid detection of any pair of SNPs in complete or high LD throughout the genome.

**Methods:**

LD, measured as the squared correlation between phased alleles (*r*^2^), can only reach a value of 1 when both loci have the same count of the minor allele. Sorting loci based on the minor allele count, followed by comparison of their alleles, enables rapid detection of loci in complete LD. Detection of loci in high LD can be optimized by computing the range of the minor allele count at another locus for each possible value of the minor allele count that can yield LD values higher than a predefined threshold. This efficiently reduces the number of pairs of loci for which LD needs to be computed, instead of considering all pairwise combinations of loci. The implemented algorithm SNPrune considered bi-allelic loci either using phased alleles or allele counts as input. SNPrune was validated against PLINK on two datasets, using an *r*^2^ threshold of 0.99. The first dataset contained 52k SNP genotypes on 3534 pigs and the second dataset contained simulated whole-genome sequence data with 10.8 million SNPs and 2500 animals.

**Results:**

SNPrune removed a similar number of SNPs as PLINK from the pig data but SNPrune was almost 12 times faster than PLINK. From the simulated sequence data with 10.8 million SNPs, SNPrune removed 6.4 and 1.4 million SNPs due to complete and high LD. Results were very similar regardless of whether phased alleles or allele counts were used. Using allele counts and multi-threading with 10 threads, SNPrune completed the analysis in 21 min. Using a sliding window of up to 500,000 SNPs, PLINK removed ~ 43,000 less SNPs (0.6%) in the sequence data and SNPrune was 24 to 170 times faster, using one or ten threads, respectively.

**Conclusions:**

The SNPrune algorithm developed here is able to remove SNPs in high LD throughout the genome very efficiently in large datasets.

**Electronic supplementary material:**

The online version of this article (10.1186/s12711-018-0404-z) contains supplementary material, which is available to authorized users.

## Background

Most current applications of genomic data involve either high-density single nucleotide polymorphism (SNP) arrays or whole-genome sequence data. Depending on the genetic diversity of the samples and the density of SNP arrays, there may be considerable redundancy in loci [1], in the sense that many pairs of SNPs are in very high or complete linkage disequilibrium (LD), i.e. they have an *r*^2^ value [2] of (close to) 1. The extent of such redundancy is expected to be especially large for genomic data from populations with a small effective population size, which indicates high levels of LD, such as that typically observed in livestock populations e.g. [1, 3]. For applications such as genomic prediction, it is common practice to remove one SNP from each pair of SNPs with an *r*^2^ value of 1 [4]. Removing loci based on high levels of pairwise LD is commonly known as LD pruning. If all possible pairs of redundant loci are considered for pruning, computation of pairwise LD between all available SNPs may not be computationally feasible.

Several tools exist that compute pairwise LD between SNPs [5, 6]. These tools are used to characterize the extent of LD in a population [7–9], to evaluate LD in regions on the genome where significant associations have been detected in genome-wide association studies (GWAS) [10–12], but also to prune for LD [5]. Characterization of the extent of LD in a population and evaluation of LD in regions on the genome require computation of LD across relatively short distances on the genome. For this reason, but also to reduce overall computational requirements, existing tools generally compute LD between pairs of SNPs located within a certain distance on the genome, as defined by the user. However, for LD pruning, it may be desirable to consider LD for all pairwise combinations of loci.

Wiggans et al. [4] noted that highly correlated SNPs should have similar MAF, and thus they evaluated only pairs of SNPs with a difference in MAF less than 2.5% units. These authors simply considered that two SNPs are perfectly correlated if the genotypes were all the same (0–0, 1–1, and 2–2) or all opposite (0–2, 1–1, and 2–0), while allowing 0.5% of the individual genotypes to differ from those rules, to allow for genotyping errors.

The objectives of our study were (1) to develop an algorithm to be able to detect quickly any pair of SNPs in complete or very high LD in very large datasets, using the assumption that highly correlated SNPs should have similar MAF, and (2) to demonstrate the performance of this algorithm.

## Methods

### Detection of SNPs in complete LD

The first step in the SNPrune algorithm is to identify redundant SNPs because they are in complete LD with other SNPs. The pseudo-code of this first step that is described hereafter is provided in Additional file 1.

One way to detect SNPs in complete LD with other SNPs, is to compute the squared correlation between their phased alleles, hereafter termed $r_{LD}^{2}$, which is equivalent to the *r*^2^ value described by Hill and Robertson [2]:

1 $$\begin{aligned} & r_{LD}^{2} \left( {a_{. ,j, .} ;a_{. ,k, .} } \right) \\ & \quad = \frac{{\left( {\left( {\mathop \sum \nolimits_{l = 1}^{2} \mathop \sum \nolimits_{i} a_{i,j,l} a_{i,k,l} } \right) - \frac{{\mathop \sum \nolimits_{l = 1}^{2} \mathop \sum \nolimits_{i} a_{i,j,l} \mathop \sum \nolimits_{l = 1}^{2} \mathop \sum \nolimits_{i} a_{i,k,l} }}{2n}} \right)^{2} }}{{\left( {\mathop \sum \nolimits_{l = 1}^{2} \mathop \sum \nolimits_{i} a_{i,j,l}^{2} - \frac{{\left( {\mathop \sum \nolimits_{l = 1}^{2} \mathop \sum \nolimits_{i} a_{i,j,l} } \right)^{2} }}{2n}} \right)\left( {\mathop \sum \nolimits_{l = 1}^{2} \mathop \sum \nolimits_{i} a_{i,k,l}^{2} - \frac{{\left( {\mathop \sum \nolimits_{k = 1}^{2} \mathop \sum \nolimits_{i} a_{i,k,l} } \right)^{2} }}{2n}} \right)}}, \\ \end{aligned}$$

where $a_{i,j,l}$ is the code of the phased allele $l$ of individual $i$ at SNP $j$, $a_{i,k,l}$ is the code of the phased allele $l$ of individual $i$ at SNP $k$, and $n$ is the number of individuals. Computation of all squared correlations between all phased alleles of $m$ loci involves computation of $\left( {m^{2} - m} \right)/2$ correlations and is computationally unfeasible if $m$ is very large, as may be the case for some high-density SNP arrays, but especially for whole-genome sequence data.

Considering that the squared correlation between phased alleles of two SNPs can only be 1 when the MAF (and thus the total count of the minor allele) are the same at both loci, a more efficient approach is to take the following steps for each of the SNPs:

1. Compute the total count of the minor allele across all individuals.
2. Sort all SNPs on these counts.
3. For any pair of SNPs that have the same minor allele count, compare the pairwise phased alleles for both haplotypes of each individual and stop as soon as an individual is identified that has a haplotype with the minor allele at one locus and the major allele at the other locus, or vice versa.
4. If all animals passed the check in step (3), then remove the “leftmost SNP” (which is the SNP with the lowest minor allele count due to the sorting in step (2); if both SNPs have the same minor allele count, the one that appeared in the data first is removed).

In step (1), first the count of the minor allele is computed for each locus $j$, by computing the sum of the alleles $(\mathop \sum \nolimits_{i} a_{i,j,l} )$ assuming that one allele is coded as 0 and the other as 1, and then translating that into the count of the number of minor alleles as:

2 $$\left\{ {\begin{array}{*{20}l} {if \left( {\mathop \sum \limits_{i} a_{i,j,l} < n + 1} \right):\mathop \sum \limits_{i} a_{i,j,l} } \\ {{else{:}}\; 2n - \mathop \sum \limits_{i} a_{i,j,l} } \\ \end{array} } \right..$$

This means that for each SNP it is assumed that the minor alleles are coded as 1 and the major alleles are coded as 0, without changing the coding of the individual alleles stored in memory. This assumption is used hereafter as well. To avoid having to change the coding for many individual allele counts for the comparison in step (3), the algorithm stores for each SNP the information on whether the count of the major or the minor allele is used. In step (3), there are two possibilities for two SNPs with the same sum of alleles:

1. Both SNPs have the same allele code for the minor allele;
2. The two SNPs have opposite allele codes for the minor allele.

If (1) is the case, then in step (3) the code of the phased alleles on each haplotype within each individual should be the same for SNPs $j$ and $k$, i.e. both should be either 0 or 1, to reach a squared correlation of 1. If (2) is the case, then in step (3) the phased alleles on each haplotype of SNPs $j$ and $k$ within each individual should either be 0 and 1 or 1 and 0, respectively, for those SNPs to reach a squared correlation of 1.

When no phased allelic data is available, the $r^{2}$ value between allele counts ($r_{ac}^{2}$) is computed, which is a good proxy for $r_{LD}^{2}$, keeping in mind that $E\left( {r_{LD}^{2} } \right) = r_{ac}^{2}$ [13]. The above steps can be applied to detect any pairs of SNPs with $r_{ac}^{2} = 1$, by replacing in Eq. (1) e.g. $\mathop \sum \nolimits_{k = 1}^{2} \mathop \sum \nolimits_{i} a_{i,j,l}$ with $\mathop \sum \nolimits_{i} x_{i,j}$, where $x_{i,j}$ is the allele count (ac) of individual $i$ at SNP $j$, yielding:

3 $$r_{ac}^{2} \left( {x_{. ,j} ;x_{. ,k} } \right) = \frac{{\left( {\mathop \sum \nolimits_{i} x_{i,j} x_{i,k} - \frac{{\mathop \sum \nolimits_{i} x_{i,j} \mathop \sum \nolimits_{i} x_{i,k} }}{n}} \right)^{2} }}{{\left( {\mathop \sum \nolimits_{i} x_{i,j}^{2} - \frac{{\left( {\mathop \sum \nolimits_{i} x_{i,j} } \right)^{2} }}{n}} \right)\left( {\mathop \sum \nolimits_{i} x_{i,k}^{2} - \frac{{\left( {\mathop \sum \nolimits_{i} x_{i,k} } \right)^{2} }}{n}} \right)}} .$$

Thus, in this case, allele counts per individual are compared instead of phased alleles within haplotypes and individuals.

### Detection of SNPs in high LD

In parallel to the identification of SNPs in complete LD, it may be of interest to identify SNPs in (very) high LD, for instance with $r_{LD}^{2}$ values higher than 0.95 or 0.99. This is the second step in the SNPrune algorithm. The pseudo-code of this second step that is described hereafter is provided in Additional file 1. Continuing from Eq. (1), i.e. using phased alleles coded as 0 or 1, the aim here is to identify pairs of SNPs with an $r_{LD}^{2}$ value higher than a pre-defined threshold $t$, for which the following holds:

4 $$\left| {r_{LD} \left( {a_{. ,j, .} ,a_{. ,k, .} } \right)} \right| > \sqrt t .$$

Note that all terms in Eq. (1) can be computed once per SNP and stored, except for $\mathop \sum \nolimits_{l = 1}^{2} \mathop \sum \nolimits_{i} a_{i,j,l} a_{i,k,l}$. To isolate this term, we use the earlier assumption that for any pair of SNPs, the minor alleles are coded as 1 and the major alleles are coded as 0. In this way, the covariance between loci arises only from combinations of alleles coded as 1 on both loci, and consequently in all cases $r_{LD}$ is higher than 0. Therefore, Eq. (4) can be simplified to:

5 $$r_{LD} \left( {a_{. ,j, .} ,a_{. ,k, .} } \right) > \sqrt t$$

Then, it follows that:

6 $$\begin{aligned} & \mathop \sum \limits_{l = 1}^{2} \mathop \sum \limits_{i} a_{i,j,l} a_{i,k,l} \\ & \quad > \sqrt {t\left( {\mathop \sum \limits_{l = 1}^{2} \mathop \sum \limits_{i} a_{i,j,l}^{2} - \frac{{\left( {\mathop \sum \nolimits_{l = 1}^{2} \mathop \sum \nolimits_{i} a_{i,j,l} } \right)^{2} }}{2n}} \right)\left( {\mathop \sum \limits_{l = 1}^{2} \mathop \sum \limits_{i} a_{i,k,l}^{2} - \frac{{\left( {\mathop \sum \nolimits_{l = 1}^{2} \mathop \sum \nolimits_{i} a_{i,k,l} } \right)^{2} }}{2n}} \right)} \\ & \quad + \frac{{\mathop \sum \nolimits_{l = 1}^{2} \mathop \sum \nolimits_{i} a_{i,j,l} \mathop \sum \nolimits_{l = 1}^{2} \mathop \sum \nolimits_{i} a_{i,k,l} }}{2n}. \\ \end{aligned}$$

Due to the assumption that minor alleles are coded as 1 and major alleles as 0, any of the sums of the products of the alleles within individual $i$, either on the same locus (i.e. $\mathop \sum \nolimits_{l = 1}^{2} \mathop \sum \nolimits_{i} a_{i,j,l}^{2}$) or between two loci (i.e. $\mathop \sum \nolimits_{l = 1}^{2} \mathop \sum \nolimits_{i} a_{i,j,l} a_{i,k,l}$), can be computed as the number of times that both alleles are 1. Note that for the observed values of the sums of allele counts, i.e. $\mathop \sum \nolimits_{l = 1}^{2} \mathop \sum \nolimits_{i} a_{i,j,l}$ for locus $j$ and $\mathop \sum \nolimits_{l = 1}^{2} \mathop \sum \nolimits_{i} a_{i,k,l}$ for locus $k$, the maximum value for $r_{LD}^{2}$ in Eq. (1) is obtained when $\mathop \sum \nolimits_{l = 1}^{2} \mathop \sum \nolimits_{i} a_{i,j,l} a_{i,k,l}$ is maximized. This is achieved, when an allele 1 at locus $j$ is as often as possible observed together with an allele 1 at locus $k$. In a formula, the expected maximum number of times that this can happen is:

7 $$\begin{aligned} & {\text{E}}\left[ {\hbox{max} \left( {\mathop \sum \limits_{l = 1}^{2} \mathop \sum \limits_{i} a_{i,j,l} a_{i,k,l} } \right)} \right] \\ & \quad = { \hbox{min} }\left( {\mathop \sum \limits_{l = 1}^{2} \mathop \sum \limits_{i} a_{i,j,l} ; \mathop \sum \limits_{l = 1}^{2} \mathop \sum \limits_{i} a_{i,k,l} } \right) \\ \end{aligned}$$

Using Eqs. (6) and (7), for any possible value of $\mathop \sum \nolimits_{l = 1}^{2} \mathop \sum \nolimits_{i} a_{i,j,l}$, we can compute and store the range of values $\mathop \sum \nolimits_{k = 1}^{2} \mathop \sum \nolimits_{i} a_{i,k,l}$ for a locus $k$ that satisfy Eq. (5). This is done by considering that the MAF of locus $j$ is lower or equal to the MAF of locus $k$. As a result, Eq. (7) simplifies to:

8 $${\text{E}}\left[ {{ \hbox{max} }\left( {\mathop \sum \limits_{l = 1}^{2} \mathop \sum \limits_{i} a_{i,j,l} a_{i,k,l} } \right)} \right] = \mathop \sum \limits_{l = 1}^{2} \mathop \sum \limits_{i} a_{i,j,l} ,$$

and Eq. (6) simplifies to:

9 $$\begin{aligned} & \mathop \sum \limits_{l = 1}^{2} \mathop \sum \limits_{i} a_{i,j,l} \\ > & \quad \sqrt {t\left( {\mathop \sum \limits_{l = 1}^{2} \mathop \sum \limits_{i} a_{i,j,l}^{2} - \frac{{\left( {\mathop \sum \nolimits_{l = 1}^{2} \mathop \sum \nolimits_{i} a_{i,j,l} } \right)^{2} }}{2n}} \right)\left( {\mathop \sum \limits_{l = 1}^{2} \mathop \sum \limits_{i} a_{i,k,l}^{2} - \frac{{\left( {\mathop \sum \nolimits_{l = 1}^{2} \mathop \sum \nolimits_{i} a_{i,k,l} } \right)^{2} }}{2n}} \right)} \\ + & \quad \frac{{\mathop \sum \nolimits_{l = 1}^{2} \mathop \sum \nolimits_{i} a_{i,j,l} \mathop \sum \nolimits_{l = 1}^{2} \mathop \sum \nolimits_{i} a_{i,k,l} }}{2n}. \\ \end{aligned}$$

For each possible value of $\mathop \sum \nolimits_{l = 1}^{2} \mathop \sum \nolimits_{i} a_{i,j,l}$, we can compute the maximum value of $\mathop \sum \nolimits_{l = 1}^{2} \mathop \sum \nolimits_{i} a_{i,k,l}$ that may still result in $\left| {r_{LD} \left( {a_{. ,j, .} ,a_{. ,k, .} } \right)} \right| > \sqrt t$. This is achieved by initializing $\mathop \sum \nolimits_{l = 1}^{2} \mathop \sum \nolimits_{i} a_{i,j,l} = 0$ and then using the following steps:

1. Increase $\mathop \sum \nolimits_{l = 1}^{2} \mathop \sum \nolimits_{i} a_{i,j,l}$ by a value of 1;
2. Set $$\mathop \sum \nolimits_{l = 1}^{2} \mathop \sum \nolimits_{i} a_{i,k,l} = \mathop \sum \nolimits_{l = 1}^{2} \mathop \sum \nolimits_{i} a_{i,j,l}$$;
3. Use Eq. (9) to determine whether the threshold can potentially be exceeded for the current values of $\mathop \sum \nolimits_{l = 1}^{2} \mathop \sum \nolimits_{i} a_{i,k,l}$ and $\mathop \sum \nolimits_{l = 1}^{2} \mathop \sum \nolimits_{i} a_{i,j,l}$;
4. If the threshold can be exceeded, then $\mathop \sum \nolimits_{l = 1}^{2} \mathop \sum \nolimits_{i} a_{i,k,l}$ is increased by 1 and repeat step (3); if not, then store $max_{k} \left( {\mathop \sum \nolimits_{l = 1}^{2} \mathop \sum \nolimits_{i} a_{i,j,l} } \right) = \mathop \sum \nolimits_{l = 1}^{2} \mathop \sum \nolimits_{i} a_{i,k,l}$, and return to step (1).

The values stored in step (4) provide the range of values of $\mathop \sum \nolimits_{l = 1}^{2} \mathop \sum \nolimits_{i} a_{i,k,l}$, and thereby the range of loci, that should be considered when evaluating high LD for a locus $j$. That is, the range of $\mathop \sum \nolimits_{l = 1}^{2} \mathop \sum \nolimits_{i} a_{i,k,l}$ values that should be considered for any value of $\mathop \sum \nolimits_{l = 1}^{2} \mathop \sum \nolimits_{i} a_{i,j,l}$ is defined as:

10 $$\mathop \sum \limits_{l = 1}^{2} \mathop \sum \limits_{i} a_{i,k,l} \in \left[ {\mathop \sum \limits_{l = 1}^{2} \mathop \sum \limits_{i} a_{i,j,l} ; max_{k} \left( {\mathop \sum \limits_{l = 1}^{2} \mathop \sum \limits_{i} a_{i,j,l} } \right)} \right].$$

In spite of the fact that this considerably reduces the number of $r_{LD}^{2}$ values that need to be computed, initial analyses showed that the total number might still be equal to several billions for datasets with a few million SNPs. In an attempt to further reduce the number of combinations for which $r_{LD}^{2}$ values need to be computed, we noted that the same principle can be applied to any (random) subset of the data. Because in our algorithm, due to sorting based on MAF, and the assumption that minor alleles are coded as 1, in all cases $\mathop \sum \nolimits_{l = 1}^{2} \mathop \sum \nolimits_{i} a_{i,k,l} \ge \mathop \sum \nolimits_{l = 1}^{2} \mathop \sum \nolimits_{i} a_{i,j,l}$. Thus, Eq. (10) can be rewritten to a restriction for $\mathop \sum \nolimits_{l = 1}^{2} \mathop \sum \nolimits_{i} a_{i,k,l}$ as:

11 $$\mathop \sum \limits_{l = 1}^{2} \mathop \sum \limits_{i} a_{i,k,l} \le max_{k} \left( {\mathop \sum \limits_{l = 1}^{2} \mathop \sum \limits_{i} a_{i,j,l} } \right).$$

Consider that we are comparing locus $j$ and $k$, for which Eq. (11) is satisfied. If for any subset of the data, Eq. (11) is not satisfied, meaning that:

12 $$\begin{aligned} & \mathop \sum \limits_{l = 1}^{2} \mathop \sum \limits_{i} a_{i, k,l} - \mathop \sum \limits_{l = 1}^{2} \mathop \sum \limits_{i} a_{i,j,l} \\ & \quad > max_{k} \left( {\mathop \sum \limits_{l = 1}^{2} \mathop \sum \limits_{i} a_{i,j,l} } \right) - \mathop \sum \limits_{l = 1}^{2} \mathop \sum \limits_{i} a_{i,j,l} \\ \end{aligned}$$

then the $r_{LD}^{2}$ value between loci $j$ and $k$ cannot exceed the threshold $t$. Thus, after identifying pairs of SNPs based on sums across all individuals using the interval defined in Eq. (10), we evaluate those pairs based on sums of subsets of the individuals. This involves computing “partial” sums for an arbitrary number of subsets of the data. We used 10 subsets that contained 10, 20,…, 100% of the data. Note that those partial sums can be computed once for every SNP and stored. Those partial sums are tested against the condition defined in Eq. (12). Whenever one of the partial sums fulfils the condition in Eq. (12), no other comparisons are performed for the pair of SNPs $j$ and $k$. Note that this comparison based on partial sums should not be performed for any pair of SNPs in which one SNP has a MAF of exactly 0.5, because in this case coding the minor allele as 1 is ambiguous. The final step of the algorithm is to compute $r_{LD}^{2}$ values for all pairs of SNPs that did not fail the test based on partial sums. In this last step, for any of those pairs of SNPs only $\mathop \sum \nolimits_{l = 1}^{2} \mathop \sum \nolimits_{i} a_{i,j,l} a_{i,k,l}$ is computed, since all other values in Eq. (1) are computed once and stored, as previously noted.

Since the algorithm to detect SNPs that are in complete LD is much more efficient than the algorithm to detect SNPs that are in high LD, both algorithms are applied sequentially in SNPrune when the aim is to prune for high LD. This is especially useful when pruning whole-genome sequence data, in which the number of SNPs in complete LD may be relatively large compared to the total number of SNPs [14].

**Fig. 1:**
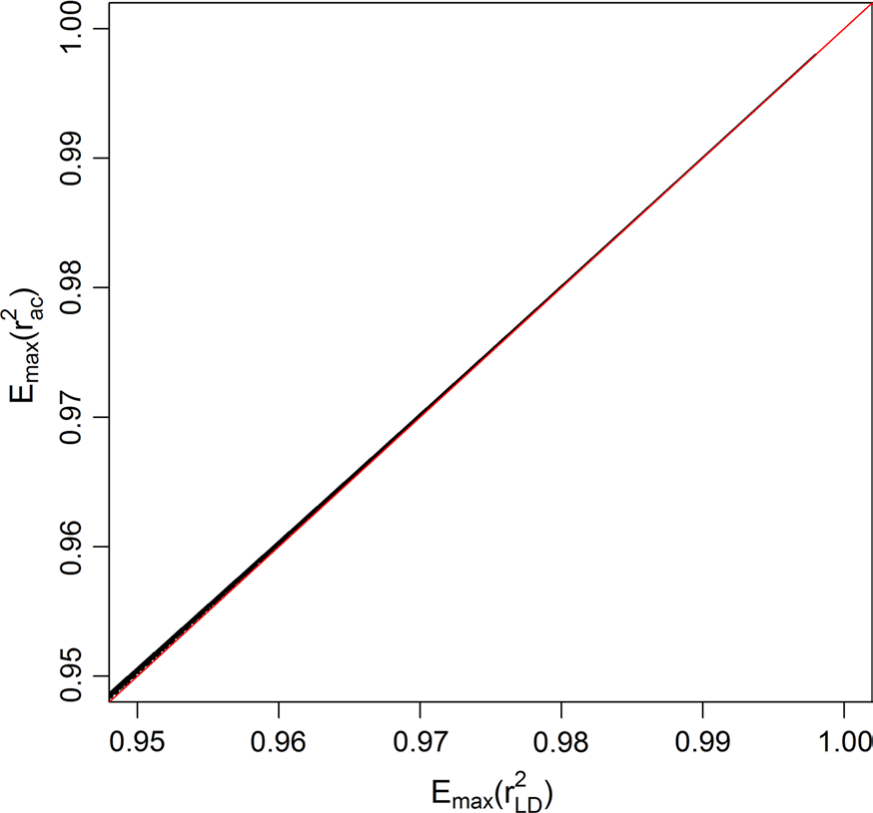
Relationship between expected maximum values for ${r}^{2}$ values computed based on allele count (${r}_{{{ac}}}^{2}$) or phased alleles (${r}_{{{LD}}}^{2}$). Pairs of $r^{2}$ values are indicated by black dots. The red line indicates $r_{{{ac}}}^{2} = r_{LD}^{2}$ as a reference

The algorithm described above, which relies on phased alleles, can be applied to allele counts as well, by assuming that, in fact, phased alleles are known. This involves the assumption that if an individual is heterozygous at two loci, the minor allele at the two SNPs reside in the same haplotype, since this will give the maximum possible contribution to the $r^{2}$ value. In Fig. 1 and Additional file 2, we show that the expected maximum values of $r_{LD}^{2}$ and $r_{ac}^{2}$ are virtually the same for values ranging from ~ 0.95 to 1.0 (i.e. $\left( {E_{max} \left( {r_{ac}^{2} } \right)/E_{max} \left( {r_{LD}^{2} } \right)} \right)$ has values ranging from 1.0000 to 1.0007), which confirms that, in this range, we can use the algorithm for $r_{LD}^{2}$ to identify which loci may surpass the threshold for $r_{ac}^{2}$. The only other change that should be made to the algorithm for using allele counts instead of phased alleles on input, is that, in the final step, $r_{ac}^{2}$ instead of $r_{LD}^{2}$ are computed.

### Data pruning

To demonstrate the performance of the algorithm developed here, we applied it to two datasets. The first dataset comprised 3534 pigs with genotypes for 52,843 SNPs, which originated from the 60k SNP array. Details of this dataset are described by Cleveland et al. [15]. The genotypes were partly imputed, and defined as a real number on the 0–2 scale. Before pruning the data, these values were transformed as follows. Values lower or equal to 0.5 were set to 0, values higher or equal to 1.5 were set to 2, and all other values were set to 1. The second dataset was simulated using QMSim [16] and contained whole-genome sequence data. The simulation process tried to mimic a historic dairy cattle population and a modern population under selection. The targeted effective population sizes ($N_{e}$) that changed over time reflected different estimated $N_{e}$ values during the history of the US and Canadian Holstein cattle [17]. After the historic population, 20 generations of 2500 animals with an equal sex ratio were simulated, using 200 males and 2500 females as parents. All male parents were replaced at each generation, and were selected based on breeding values for a polygenic trait with an accuracy of 0.8. After each generation, half of the female parents were replaced by all females generated in this generation. The final dataset, used for analyses, contained all 2500 animals of the last generation. For these animals, 10,812,225 segregating SNPs were available, spread across 29 autosomes. The phase of the alleles was outputted by QMSim and assumed to be known without error in the analyses to prune SNPs based on LD.

The algorithm developed here was used to prune both datasets that are described above based on $r_{ac}^{2}$. In addition, the whole-genome sequence dataset was also pruned based on phased alleles, to demonstrate the difference between both strategies. In all cases, a threshold for $r^{2}$ of 0.99 was used. To compare performances, the datasets were also pruned based on $r_{ac}^{2}$ using the software PLINK version 1.90 beta [18, 19]. Pruning based on LD in PLINK is performed using a sliding window along the genome. For the pig dataset, it was still possible to consider all pairwise combinations by including all SNPs in one window. For the sequence data, this was not possible within acceptable computing time. Thus, sliding windows of 50, 500, 5000, 50,000, 500,000, or 5,000,000 SNPs were used for the sequence data, that were shifted forward in steps of 10% of the window size, i.e. with 5, 50, 500, 5000, 50,000, or 500,000 SNPs, respectively. The LD threshold used for pruning was 0.99, i.e. the same value as for SNPrune. For window sizes of up to 500,000 SNPs, PLINK was also run using the option that estimates $r_{LD}^{2}$ based on phasing information obtained with maximum likelihood. The command line argument used to run PLINK using $r_{ac}^{2}$ was (using a window of 5000 SNPs) the following: plink --bed data.bed --indep-pairwise 5000 500 0.99, and when using the maximum likelihood phasing information it was: plink --bed data.bed --indep-pairphase 5000 500 0.99.

## Results

### Pig data

The pig dataset was pruned only by considering allele counts, since no map or phasing information was available. We performed pruning three times by using the two algorithms SNPrune and PLINK (Table 1). The first time, the data were fed to both programs without any pre-sorting based on MAF, and SNPrune and PLINK removed 9126 and 9038 SNPs, respectively, with 6792 SNPs that overlapped. This relatively small overlap was most likely largely due to the removal of different SNPs from each pair in high LD, because the order in which SNPs were processed differed between programs. The second time, the order of the SNPs was based on increasing MAF, to make sure that both programs processed the SNPs in the same order. In this case, SNPrune and PLINK removed 9126 and 9098 SNPs, respectively, with 6844 SNPs that overlapped. Finally, the data were first pre-sorted on MAF, and then pre-pruned for complete LD. This pruning step removed 3830 SNPs. The remaining 49,013 SNPs, still pre-sorted based on MAF, were pruned for high LD. In this case, SNPrune and PLINK removed 5296 and 5283 SNPs, respectively, with 4939 SNPs that overlapped.

**Table 1:** Number of pruned SNPs from the 52,843 SNPs present on the 60k pig SNP array

Analysis	One step^a^	One step^a^ pre-sorted on MAF	Two step^a^ pre-sorted on MAF	
Number of SNPs pruned out	Total	Total	High LD	Total^b^
SNPrune	9126	9126	5296	9126
PLINK	9038	9098	5283	9113
Overlap^c^	6792	6844	4939	8778

^a^Either the LD pruning is done in one step, or in two steps, where 3830 SNPs in complete LD with other SNPs are removed in the first step, and the remaining SNPs in high LD are removed in the second step

^b^Including the 3830 removed due to complete LD

^c^Overlap between SNPs pruned by SNPrune and PLINK

PLINK computed all 1.4 × 10^9^ pairwise $r_{ac}^{2}$ values. Considering the analyses of SNPrune, a relevant question is how efficient is the algorithm in avoiding computation of $r_{ac}^{2}$ values that were predicted to never reach the imposed threshold of 0.99. Using Eq. (9), SNPrune filtered 9,898,092 pairs of SNPs that potentially could have a $r_{ac}^{2}$ higher than 0.99 (Table 2). Based on partial sums of the minor allele, the number of $r_{ac}^{2}$ that needed to be computed decreased to 3,718,230. This was only 0.27% of the total number of possible $r_{ac}^{2}$ values in the entire dataset, or 0.31% of the total number of possible $r_{ac}^{2}$ values after pruning for complete LD.

**Table 2:** Numbers of SNP pairs for which *r*^*2*^ values were computed for the pig data

Number of pairs of SNPs	Allele counts
Possibly > 0.99	9,898,092
< 0.99 (partial sums)	6,179,862
Computed $r^{2}$ values	3,718,230
Percentage (all SNPs)^a^	0.27
Percentage (after pruning for complete LD)^b^	0.31

^a^Number of computed $r^{2}$ values as percentage of the total number of *r*^*2*^ values

^b^Number of computed $r^{2}$ values as percentage of the total number of *r*^*2*^ values after pruning for complete LD

### Simulated sequence data

First, pruning of the simulated sequence data was done by using SNPrune based on phased alleles or allele counts (Table 3). Results using one or ten threads were identical. In total, 6,367,210 and 6,366,971 SNPs were removed based on complete LD, and another 1,428,122 and 1,428,725 SNPs were removed based on an $r_{ac}^{2}$ or $r_{LD}^{2}$ higher than 0.99, using allele counts and phased alleles, respectively. In total, 3,016,893 and 3,016,529 of 10,812,225 SNPs remained after pruning, using allele counts or phased alleles, respectively. Sets of SNPs that were removed by using phased alleles or allele counts showed an overlap of more than 99.9% (results not shown).

**Table 3:** Number of pruned SNPs from the 10,812,225 SNPs included in the simulated sequence dataset

Pruning approach	Number of SNPs pruned out			Number of SNPs left
	Complete LD	High LD	Total	
SNPrune allele counts	6,367,210	1,428,122	7,795,332	3,016,893
SNPrune phased alleles	6,366,971	1 428 725	7,795,696	3,016,529
PLINK (w50^a^) NP^b^	NA	NA	5,401,197	5,411,028
PLINK (w500^a^) NP^b^	NA	NA	7,547,118	3,265,107
PLINK (w5000^a^) NP^b^	NA	NA	7,740,937	3,071,288
PLINK (w50000^a^) NP^b^	NA	NA	7,750,558	3,061,667
PLINK (w500000^a^) NP^b^	NA	NA	7,752,008	3,060,217
PLINK (w5000000^a^) NP^b^	NA	NA	7,752,834	3,059,391
PLINK (w50^a^) MLP^c^	NA	NA	5,401,527	5,410,698
PLINK (w500^a^) MLP^c^	NA	NA	7,543,234	3,268,991
PLINK (w5000^a^) MLP^c^	NA	NA	7,741,279	3,070,946
PLINK (w50000^a^) MLP^c^	NA	NA	7,751,008	3,061,217
PLINK (w500000^a^) MLP^c^	NA	NA	7,752,485	3,059,740

^a^Using a sliding window of 50, 500, 5000, 50,000, 500,000 or 5,000,000 SNPs

^b^The $r^{2}$ values are computed between allele counts, considering no phasing (NP)

^c^The $r^{2}$ values are computed between alleles that are phased based on maximum likelihood phasing (MLP)

Pruning of the sequence data was also done using PLINK. It was not possible to consider all possible pairwise $r^{2}$ values within an acceptable computing time, so we used sliding windows ranging from 50 to 5,000,000 SNPs. Whether pruning was based on allele counts or maximum likelihood derived phasing information, hardly affected the results. For example, the difference in number of SNPs removed for the window size of 5000 SNPs was only 342, which represents 0.0044% of the SNPs removed, while the overlap between removed SNPs was more than 99.9%. This result is in agreement with the SNPrune results using either allele counts or phased alleles, for which the overlap between removed SNPs was also close to 100%. When allele counts were used in PLINK, 5,411,028 SNPs remained after pruning with a window of 50 and this number dropped to 3,059,391 with a window of 5,000,000 (Table 3). When a window of 5,000,000 SNPs was used, PLINK removed 42,498 SNPs less than SNPrune based on allele counts.

The number of $r^{2}$ values computed by PLINK ranged from 2.65 × 10^9^ to 2.65 × 10^14^ with window sizes ranging from 50 to 5,000,000 (Table 4). SNPrune identified ~ 107.5 × 10^9^ pairs of SNPs that potentially could have a $r_{ac}^{2}$ higher than 0.99 (Table 5). Based on partial sums of the minor allele, the number of $r_{ac}^{2}$ that needed to be computed was reduced to ~ 61.2 × 10^9^. Thus, the number of computed $r^{2}$ values was only 0.08% of the total number of possible $r^{2}$ values in the entire dataset, or 0.47% of the total number of possible $r^{2}$ values after pruning for complete LD.

**Table 4:** Number of computed *r*^*2*^ values in the simulated sequence dataset using PLINK

Window size	Step size	Number of computed *r*^*2*^ values^a^
50	5	2.65 × 10^9^
500	50	2.70 × 10^10^
5000	500	2.70 × 10^11^
5000	5000	2.70 × 10^12^
500,000	50,000	2.70 × 10^13^
5,000,000	500,000	2.70 × 10^14^

^a^Computed as $\left( {10,812,225/ss} \right) \times \left( {ws^{2} - ws} \right)/2$, where 10,812,225 is the total number of SNPs, $ss$ is the step size (i.e. the size of the shift of the windows), and $ws$ is the window size used

**Table 5:** Number of pairs of SNPs for which *r*^*2*^ values were computed for the simulated sequence dataset

Number of pairs of SNPs	Phased alleles	Allele counts
Possibly > 0.99	107,576,540,902	107,567,702,834
< 0.99 (partial sums)	61,142,300,573	61,152,664,161
Computed $r^{2}$ values	46,434,240,329	46,415,038,673
Percentage (all SNPs)^a^	0.08	0.08
Percentage (after pruning for complete LD)^b^	0.47	0.47

^a^The number of computed $r^{2}$ values as percentage of the total number of *r*^*2*^ values

^b^The number of computed $r^{2}$ values as percentage of the total number of *r*^*2*^ values after pruning for complete LD

### Computational requirements

**Fig. 2:**
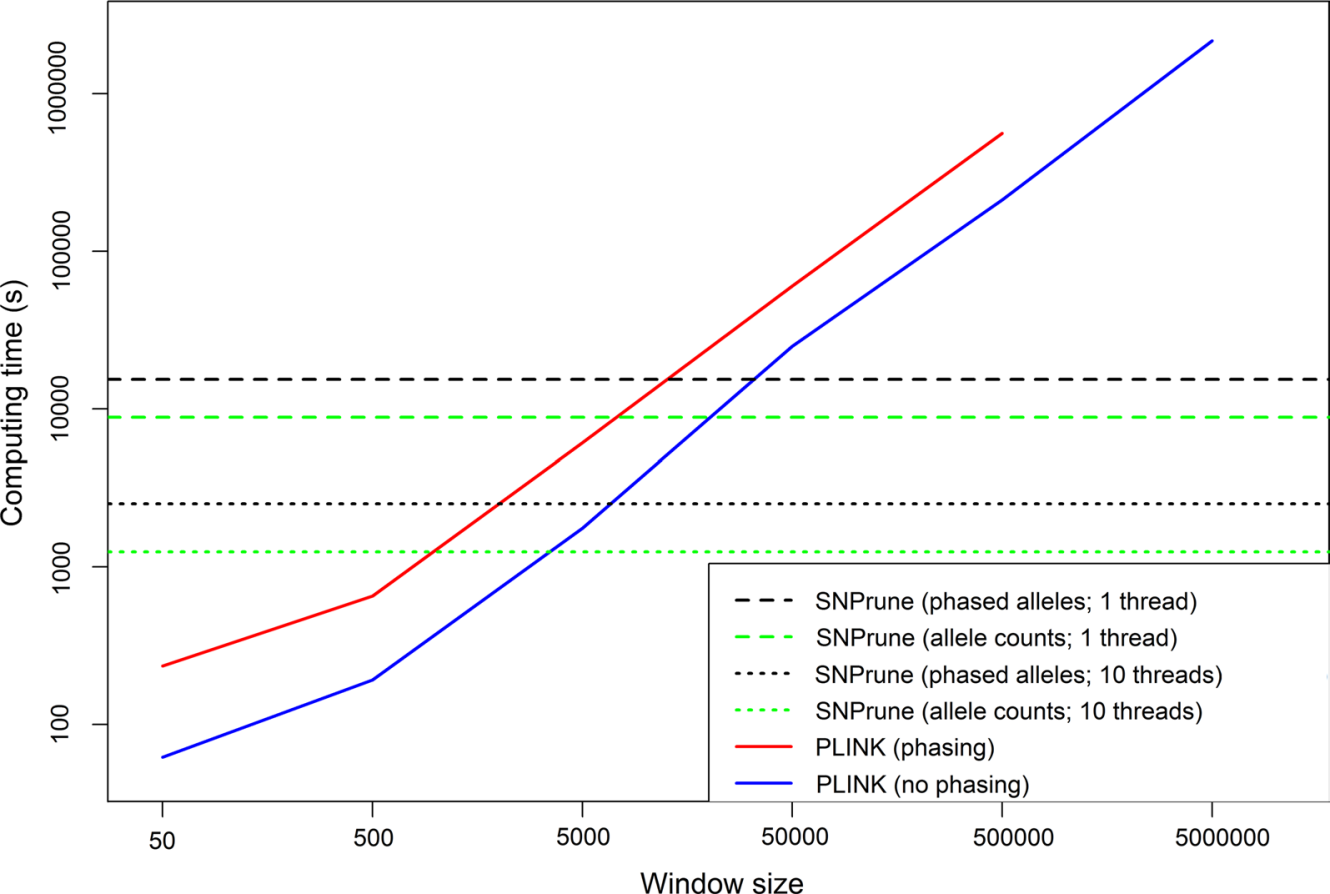
Computation time to prune the sequence data using SNPrune and PLINK with various settings

The computing time required to process the pig data, averaged across five analyses and using one thread for all analyses, was equal to 17.2 s for SNPrune and 3 min 9.3 s for PLINK. Thus, SNPrune was 11 times faster than PLINK. For the sequence data with a single thread, SNPrune required 4 h 16 min using phased alleles, and 2 h 28 min using allele counts (Fig. 2). Using ten threads, SNPrune required 42 min using phased alleles, and 21 min using allele counts, i.e. the latter almost halved the computing time. Computing times for PLINK were as short as only 1 min with a sliding window of 50, and increased linearly with increasing window size, to a computing time of 58 h 44 min using a window size of 500,000 SNPs. Window size and step size (i.e. the distance between the first SNP for two consecutive windows) increased each time by a factor 10. This means that when the window size increased by a factor 10, the number of computed $r^{2}$ values roughly increased by 10^2^, while the number of windows decreased by a factor 10. Thus, in effect, the number of computations increased by a factor ~ 10, which caused the observed linear increase with increasing window size.

For the pig data, the peak RAM use was 213 Mb for SNPrune, and 215 Mb for PLINK. For the sequence data, the peak RAM use for SNPrune was 56.5 Gb using phased alleles and 29.8 Gb using allele counts. We ran the analysis using allele counts of all SNPs in PLINK (i.e. using a window size comprising the entire genome) for some time, but we did not let the analysis finish since it was too long. However, this allowed us to assess that the peak memory used in this case by PLINK was 33.0 Gb, but the memory use of PLINK dropped considerably, when smaller windows sizes were used.

## Discussion

In this paper, we describe the algorithm SNPrune, which we developed to efficiently prune SNPs in complete or very high LD in datasets containing a large number of SNPs. SNPrune was 11 times faster than PLINK when applied to a dataset with 3534 pigs and 52k SNPs. We also show that SNPrune is able to prune sequence data with 2500 individuals and more than 10 million SNPs in 21 min using 10 threads. These results demonstrate that SNPrune is a very efficient algorithm to prune large datasets for high levels of LD, e.g. $r^{2} > 0.99$, as used in our study.

Pruning based on phased alleles versus allele counts gave similar results, both with SNPrune and PLINK, but when the analysis used allele counts, it was considerably faster, for both algorithms. For SNPrune, the analysis using allele counts was almost twice as fast compared to using phased alleles, while for PLINK the analysis using allele counts was ~ 2 to 4 times faster. The explanation of this factor 2 found for SNPrune is that the highest cost of SNPrune is to compute the cross-products of either the genotypes or the phased alleles, for all pairs of SNPs for which the $r^{2}$ is computed. When using phased alleles, each of those cross-products involves twice as many multiplications compared to when using allele counts. For PLINK, the difference is larger, because PLINK actually performs the phasing itself, in addition to computing the $r^{2}$ values. Interpolating the results in Fig. 2 shows that for window sizes of ~ 15,000 and ~ 25,000 SNPs, PLINK needs the same computing time for using either allele counts or phased alleles, respectively. In both cases, SNPrune removed only ~ 0.4% more SNPs than PLINK. Thus, in the case of a sequence dataset with similar properties as that used in our study, using PLINK with a sliding window of ~ 20,000 SNPs achieves similar results as SNPrune in similar computing time, albeit that PLINK still leaves some pairs of distant SNPs in high LD in the data.

### Applications of SNPrune

**Fig. 3:**
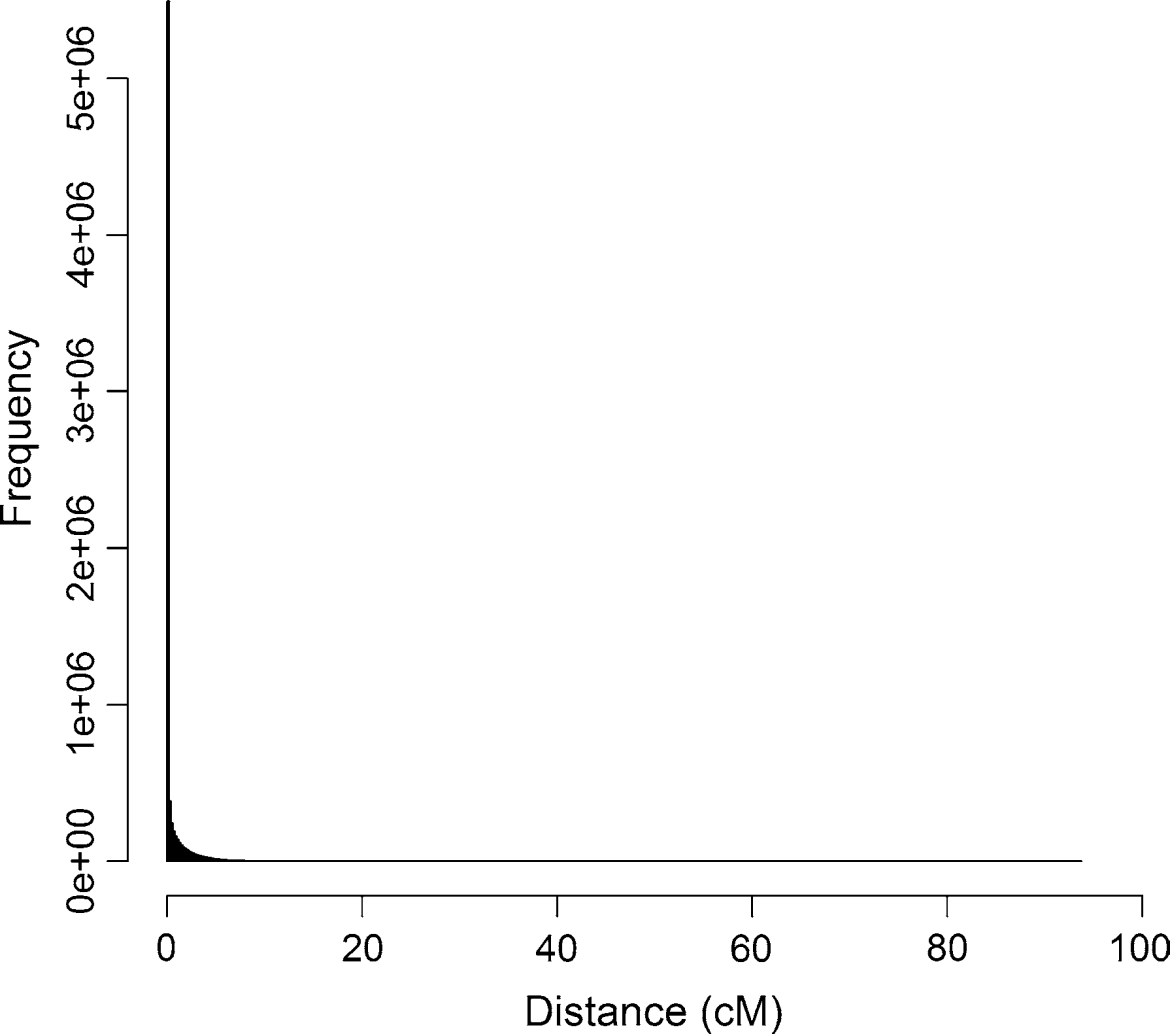
Distribution of distances between pairs of SNPs pruned from the sequence data that were located on the same chromosome

To limit computing time with PLINK or other similar software, usually only SNPs in close proximity on the same chromosome, e.g. less than 2 Mb apart, are compared. When the goal is to evaluate levels of LD, such a window-based approach is usually sufficient. When the goal is to reduce co-linearity between loci, for instance to improve the performance of a subsequent genomic prediction model, then it is desirable to consider all possible pairwise combinations of loci. SNPrune enables the detection of pairs of SNPs that are in strong LD regardless of their locations in the genome. In randomly mating populations, LD between loci on different chromosomes is expected to be low, and pairwise high levels of LD may only appear by chance. However, high levels of LD between loci on different chromosomes may be more frequent in highly structured populations such as livestock populations. Our simulated data mimicked a dairy cattle population under selection. Among the removed SNPs, considering the analyses based on allele counts, 0.6% belonged to a pair of SNPs that were located on different chromosomes. The remaining 99.4% belonged to a pair of SNPs that were located on the same chromosome, and were on average 0.59 cM apart. Nevertheless, pairs of SNPs that were on the same chromosome could be separated by a large distance (Fig. 3).

In the context of genomic prediction, high LD between SNPs can impair model performance [14]. The aim of genomic prediction is to put most of the emphasis, in terms of estimated effects, on SNPs in close LD with typically unobserved causal variants. In this sense, “spurious” associations, in which a SNP has a large estimated effect due to high LD with a causal variant although it is not close to it on the genome, are not desired. Such spurious associations will lead to estimated SNP effects that erode quickly over time. Thus, from the perspective of genomic prediction, it is important to consider all pairwise combinations of SNPs when pruning for high LD, rather than only those in a sliding window, as for instance in VanRaden et al. [20]. For the removal of spurious associations, the choice of which SNP should be removed from a pair of SNPs that are in high LD, could perhaps be made in a more sophisticated way. For a pair of distantly located SNPs with high LD, one option is to retain the SNP that has the highest LD with the surrounding SNPs. If the pair of SNPs is in LD with a causal variant, then this SNP is expected to be more likely physically closely located to this variant.

Pruning for LD may considerably reduce the computational burden of genomic prediction based on sequence data, since, in our study, the number of SNPs in the simulated sequence data decreased by 72%. In the literature, a reduction of 58% was reported when imputed sequence data were used with 14 million SNPs for 5553 Holstein–Friesian dairy bulls and LD pruning in subsets of the SNPs was applied [14], and in another study, a reduction of 99.5% was observed using 145 tomato accessions with imputed sequence data with 19.6 million SNPs [21]. Nevertheless, our results obtained with PLINK and the distribution of the distance between pairs of SNPs exceeding the $r^{2}$ threshold (Fig. 3) show that using a window approach does lead to the removal of the majority of SNPs that are in high LD with each other.

The ability to efficiently identify SNPs that are in high LD with other SNPs located elsewhere in the genome, while they are in low LD with the surrounding SNPs, is also useful for other applications. In empirical analyses, high LD of one SNP with a group of SNPs located on another chromosome, maybe an indication that this SNP resides in a misassembled segment of the reference genome [22]. Therefore, SNPrune could be also a very useful tool to detect rapidly genome segments that may be misassembled. Similarly, the algorithm could be used in LD-based approaches to derive the chromosomal locations of unmapped SNPs [23–25].

### Fine-tuning SNPrune

The amount of whole-genome sequence data generated is rapidly growing and soon, datasets for 100,000 sequenced animals may be available. Computing time is expected to increase linearly with increasing numbers of individuals, both for PLINK and SNPrune, because the number of multiplications required to compute a single $r^{2}$ value is proportional to the number of individuals. In the analyses of the simulated sequence data, 57% of the pairs of SNPs that were identified based on their MAF as possibly exceeding the threshold of 0.99, were discarded by evaluating partial sums (i.e. sums based on subsets of the data) of the minor allele rather than the sums of the minor allele of the entire data. Here, we used ten partial sums, comprising 10, 20,…, 100% of the data. With more individuals, it is possible that the percentage of pairs of SNPs that are discarded based on partial sums will be larger, and fine-tuning the number of subsets may increase this percentage even more.

The implementation of SNPrune presented in this paper is not able to deal with missing genotypes, but we showed that it is able to efficiently remove SNPs in high LD. Pruning for considerably lower LD thresholds, i.e. $r^{2}$ values lower than 0.8, means that the maximum difference in MAF for a pair of SNPs to possibly exceed this threshold will be considerably larger. This could lead to a relatively small reduction in the number of $r^{2}$ values that need to be computed, compared to all pairwise combinations. Extending the algorithm to tolerate small amounts of missing data, and fine-tuning its performance for considerably lower LD thresholds, may increase its potential for other applications than those investigated in our study.

While SNPrune is able to remove SNPs in high LD throughout the genome in large datasets very efficiently, the current implementation uses a one-byte format to handle (phased) SNP genotypes. Therefore, additional computing improvements could be realised by using a packed 2-bit format, which will allow bit-level operations and parallelism, as detailed by Chang et al. [18]. Adaptation of the two algorithms for bit-level parallelism is possible because their main operations involve integers 0 and 1 for phased genotypes, or integers 0, 1, and 2 for allele counts (if missing values are ignored). As previously mentioned, allele counts could be considered as phased SNPs. Using such a packed 2-bit format, bit-level operations will improve the computation of terms such as $\mathop \sum \nolimits_{l = 1}^{2} \mathop \sum \nolimits_{i} a_{i,j,l}$ or $\mathop \sum \nolimits_{l = 1}^{2} \mathop \sum \nolimits_{i} a_{i,j,l} a_{i,k,l}$, and reduce RAM and CPU time requirements, which would improve the efficiency of SNPrune even more.

## Conclusions

We developed an algorithm SNPrune that is able to remove SNPs in high LD throughout the genome in large datasets very efficiently. For a simulated whole-genome sequence dataset, we show that 72% of the SNPs were removed by pruning SNPs with $r^{2}$ higher than 0.99, which reduces computational burden in subsequent genomic prediction due to the steeply reduced dimension of the data, but also to the considerable reduction in co-linearity in the SNP data. The SNPrune algorithm may also be useful for other applications such as detection of misassembled segments in reference genomes.

## Additional files

**Additional file 1.** Pseudo code for the algorithms presented.

**Additional file 2.** Relationship between expected maximum values for $\varvec{r}_{{\varvec{LD}}}^{2}$ and $\varvec{r}_{{\varvec{ac}}}^{2}$.
