# Supplementary material for: SNPrune: an efficient algorithm to prune large SNP array and sequence datasets based on high linkage disequilibrium
Source: Genet Sel Evol. 2018 Jun 26;50:34. doi: 10.1186/s12711-018-0404-z (PMC6019535; doi:10.1186/s12711-018-0404-z)
Supplement: Supplementary file 1 — Additional file 1. Pseudo code for the algorithms presented. [file 12711_2018_404_MOESM1_ESM.docx]

### Additional file 1 – Pseudo code for the presented algorithms

Type of file: docx

## Detection of SNP in complete LD

#

!Step 1

For all SNPs;

Compute the total count of the minor allele

!Step 2

Sort all SNPs based on increasing counts of the minor allele, and store whether 0 or 1 is the minor allele

!Step 3

For all SNPs, following the sorted list from step 2;

3a) Compare the minor allele count with the following SNPs, until a SNP with a higher count is detected

3b) For SNPs with an equal minor allele count, starting with the “leftmost” pair, compare all alleles or allele counts until

- If the minor allele is coded the same; an individual with different alleles or allele counts is found.
- Else if the minor allele is coded differently; an individual with a difference between alleles other than 1 is found (when using alleles), or an individual with a sum of the allele counts at both loci other than 2 is found (when using allele counts)

3c) If no such individuals are observed, the alleles/allele counts are identical at both loci and the “leftmost” SNP is removed.

3d) Proceed with the next SNP

## Detection of SNP in high LD

#

!Step 1

For all SNPs;

Compute the total count of the minor allele

!Step 2

Sort all SNPs based on increasing counts of the minor allele, and store whether 0 or 1 is the minor allele

!Step 3

For each possible count of the minor allele, determine the maximum count of the minor allele at another loci that still may yield an $r_{LD}^{2}$ value greater than the defined threshold.

!Step 4

For all SNPs, following the sorted list from step 2;

4a) Compare the minor allele count of the following SNPs with maximum count of the minor allele at another loci that still may yield an $r_{LD}^{2}$ value greater than the defined threshold. For each SNP where this applies, starting with the “leftmost” pair;

- Compute the $r_{LD}^{2}$ (or $r_{ac}^{2}$) value with this SNP
- If the $r_{LD}^{2}$ (or $r_{ac}^{2}$) value exceeds the threshold, then remove the leftmost SNP.

3c) If no such individuals are observed, the “leftmost” SNP is removed.

3d) Proceed with the next SNP
