# Supplementary material for: SNPrune: an efficient algorithm to prune large SNP array and sequence datasets based on high linkage disequilibrium
Source: Genet Sel Evol. 2018 Jun 26;50:34. doi: 10.1186/s12711-018-0404-z (PMC6019535; doi:10.1186/s12711-018-0404-z)
Supplement: Supplementary file 2 — Additional file 2. Relationship between expected maximum values for $\varvec{r}_{{\varvec{LD}}}^{2}$rLD2 and $\varvec{r}_{{\varvec{ac}}}^{2}$rac2. [file 12711_2018_404_MOESM2_ESM.docx]

### Additional file 2 – The relationship between expected maximum values for $\boldsymbol{r}_{\boldsymbol{LD}}^{\boldsymbol{2}}$ and $\boldsymbol{r}_{\boldsymbol{ac}}^{\boldsymbol{2}}$

Type of file: docx

Here we use a slightly different notation than in the main text. Consider that $p_{j}$ is the frequency of the minor allele at locus *j*, $p_{k}$ is the frequency of the minor allele at locus *k*, minor alleles are coded as 1, and major alleles are coded as 0. Then, considering phased alleles, the variance for e.g. locus *j* is $p_{j}\left( 1-p_{j} \right)$. And the covariance between loci *j* and *k* is, considering that e.g. $f\left( 11 \right)$ is the frequency of haplotype 11 and $a_{. ,j, .}$ is the allele at locus *j*:

$$cov\left( a_{. ,j, .},a_{. ,k, .} \right)=E\left[ \left( a_{. ,j, .}a_{. ,k, .}-E\left[ a_{. ,j, .} \right]E\left[ a_{. ,k, .} \right] \right) \right]=\left( f\left( 0 \right)*0^{2}+f\left( 01 \right)*0*1+f\left( 10 \right)*1*0+f\left( 11 \right)*1^{2}-p_{j}p_{k} \right)=\left( f\left( 11 \right)-p_{j}p_{k} \right)$$

The expected maximum value of $f\left( 11 \right)$, considering that we process the SNPs such that $p_{j}\leq p_{k}$, is obtained when all alleles 1 at locus *j* are accompanied by an allele 1 at locus *k*:

$$E_{max}\left( f\left( 11 \right) \right) =\min\left( p_{j},p_{k} \right)=p_{j}$$

Thus, the maximum expected value for the covariance is:

$$E_{max}\left( cov\left( a_{. ,j, .},a_{. ,k, .} \right) \right)=max\left( f\left( 11 \right)-p_{j}p_{k} \right)=\max\left( f\left( 11 \right) \right)-p_{j}p_{k}=\left( p_{j}-p_{j}p_{k} \right)=p_{j}\left( 1-p_{k} \right)$$

Thus, the maximum expected $r_{LD}^{2}$ value is:

$$E_{max}\left( r_{LD}^{2} \right)=\frac{\left( p_{j}\left( 1-p_{k} \right) \right)^{2}}{p_{j}\left( 1-p_{j} \right)p_{k}\left( 1-p_{k} \right)}=\frac{p_{j}\left( 1-p_{k} \right)}{p_{k}\left( 1-p_{j} \right)}$$

A similar reasoning can be used for $r_{ac}^{2}$. In this case, we assume that the loci are in Hardy-Weinberg equilibrium. So, the variance for e.g. locus *j* is $2p_{j}\left( 1-p_{j} \right)$. And the covariance between loci *j* and *k* is, noting that e.g. $f_{ac}\left( 21 \right)$ is the frequency of observing allele count 2 at locus *j* and allele count 1 at locus *k*, and considering that $x_{. ,j}$ is the allele count at locus *j*:

$$cov\left( x_{. ,j},x_{. ,k} \right)=E\left[ \left( x_{. ,j}x_{. ,k}-E\left[ x_{. ,j} \right]E\left[ x_{. ,k} \right] \right) \right]=\left( f_{ac}\left( 22 \right)*2*2+f_{ac}\left( 21 \right)*2*1+f_{ac}\left( 12 \right)*1*2+ f_{ac}\left( 11 \right)*1*1+f_{ac}\left( 10 \right)*1*0+f_{ac}\left( 01 \right)*0*1+f_{ac}\left( 00 \right)*0*0-2p_{j}2p_{k} \right)=\left( f_{ac}\left( 22 \right)*4+f_{ac}\left( 21 \right)*2+f_{ac}\left( 12 \right)*2+ f_{ac}\left( 11 \right)*1-2p_{j}2p_{k} \right)$$

To obtain the maximum value of this covariance, the number of appearances of allele counts of 2 at both loci, or equivalently $f_{ac}\left( 22 \right)$, needs to be maximized. Given that we assume Hardy-Weinberg equilibrium and $p_{j}\leq p_{k}$, this means that all allele counts 2 at locus *j* should be accompanied by an allele count of 2 at locus *k*. Thereafter, considering the remaining number of allele counts of 2 at locus *k*, the number of appearances of allele counts 1 at locus *j* and 2 at locus *k*, or equivalently $f_{ac}\left( 12 \right)$, needs to be maximized. Finally, the number of appearances of allele counts of 1 at both loci, or equivalently $f_{ac}\left( 11 \right)$, needs to be maximized. Expressions for the frequencies of the combinations of allele counts that make a contribution to the maximum possible $cov\left( x_{. ,j},x_{. ,k} \right)$ are obtained as follows:

$$max\left( f_{ac}\left( 22 \right) \right)=max\left( p_{j}^{2},p_{k}^{2} \right)=p_{j}^{2}$$

Then, $f_{ac}\left( 21 \right)=0$, and,

$$max\left( f_{ac}\left( 12 \right) \right)=min\left( f_{ac}\left( 1. \right);f_{ac}\left( .2 \right)-f_{ac}\left( 22 \right) \right)=min\left( 2p_{j}\left( 1-p_{j} \right);p_{k}^{2}-p_{j}^{2} \right)$$

Finally, there will be only allele counts 1 left on locus *j* if these were not all used to maximize $f_{ac}\left( 12 \right)$. This is the case when:

$$2p_{j}\left( 1-p_{j} \right)>p_{k}^{2}-p_{j}^{2}$$

$$2p_{j}\left( 1-p_{j} \right)-p_{k}^{2}+p_{j}^{2}>0$$

$$2p_{j}-p_{j}^{2}-p_{k}^{2}>0$$

Such that:

$$max\left( f_{ac}\left( 11 \right) \right)=max\left( 2p_{j}-p_{j}^{2}-p_{k}^{2};0 \right)$$

An overview of these expressions is given in Table S1.

Table S1. Required frequencies for different combinations of allele counts at loci j and k, to maximize $r_{ac}^{2}$between those two loci.

| Locus |  | *k* |  |
| --- | --- | --- | --- |
|  | Allele count | 2 | 1 |
| *J* | 2 | $p_{j}^{2}$ | 0 |
|  | 1 | $min\left( 2p_{j}(1-p_{j});p_{k}^{2}-p_{j}^{2} \right)$ | $max\left( 2p_{j}-p_{j}^{2}-p_{k}^{2};0 \right)$ |

Considering these expressions,

$$E_{max}\left( r_{ac}^{2} \right)=\frac{\left( 4p_{j}^{2}+2*min\left( 2p_{j}\left( 1-p_{j} \right);p_{k}^{2}-p_{j}^{2} \right)+max\left( 2p_{j}-p_{j}^{2}-p_{k}^{2};0 \right) \right)^{2}}{2p_{j}\left( 1-p_{j} \right){2p}_{k}\left( 1-p_{k} \right)}$$

Values of $E_{max}\left( r_{LD}^{2} \right)$ and $E_{max}\left( r_{ac}^{2} \right)$ were numerically compared in Figures S1 and S2 by simply plotting their values against each other, considering that, given the coding of the minor alleles as 1, $p_{j}\in[0,0.5]$, $p_{k}\in[0,0.5]$ and $p_{j}\leq p_{k}$. These figures show that for $r_{LD}^{2}\geq0.95$, $E_{max}\left( r_{ac}^{2} \right)\approx E_{max}\left( r_{LD}^{2} \right)$. Combining the above expressions for $E_{max}\left( r_{LD}^{2} \right)$ and $E_{max}\left( r_{ac}^{2} \right)$, considering that $p_{k}\left( 1-p_{j} \right)=\frac{p_{j}\left( 1-p_{k} \right)}{E_{max}\left( r_{LD}^{2} \right)}$, we obtain:

$$E_{max}\left( r_{ac}^{2} \right)=\frac{\left( 4p_{j}^{2}+2*min\left( 2p_{j}\left( 1-p_{j} \right);p_{k}^{2}-p_{j}^{2} \right)+max\left( 2p_{j}-p_{j}^{2}-p_{k}^{2};0 \right) \right)^{2}}{\frac{4p_{j}^{2}\left( 1-p_{k} \right)^{2}}{E_{max}\left( r_{LD}^{2} \right)}}=\frac{\left( 4p_{j}^{2}+2*min\left( 2p_{j}\left( 1-p_{j} \right);p_{k}^{2}-p_{j}^{2} \right)+max\left( 2p_{j}\left( 1-p_{j} \right)-p_{k}^{2}+p_{j}^{2};0 \right) \right)^{2}}{4p_{j}^{2}\left( 1-p_{k} \right)^{2}}E_{max}\left( r_{LD}^{2} \right)$$

Applying this expression (see Figure S3), shows that for $E_{max}\left( r_{LD}^{2} \right)\geq0.95$, $E_{max}\left( r_{ac}^{2} \right)$ is at most ~1.0007 times bigger than $E_{max}\left( r_{LD}^{2} \right)$. For $E_{max}\left( r_{LD}^{2} \right)\geq0.99$, $E_{max}\left( r_{ac}^{2} \right)$ is at most ~1.00003 times bigger than $E_{max}\left( r_{LD}^{2} \right)$, so in this case, we can assume that $E_{max}\left( r_{ac}^{2} \right)=E_{max}\left( r_{LD}^{2} \right)$.


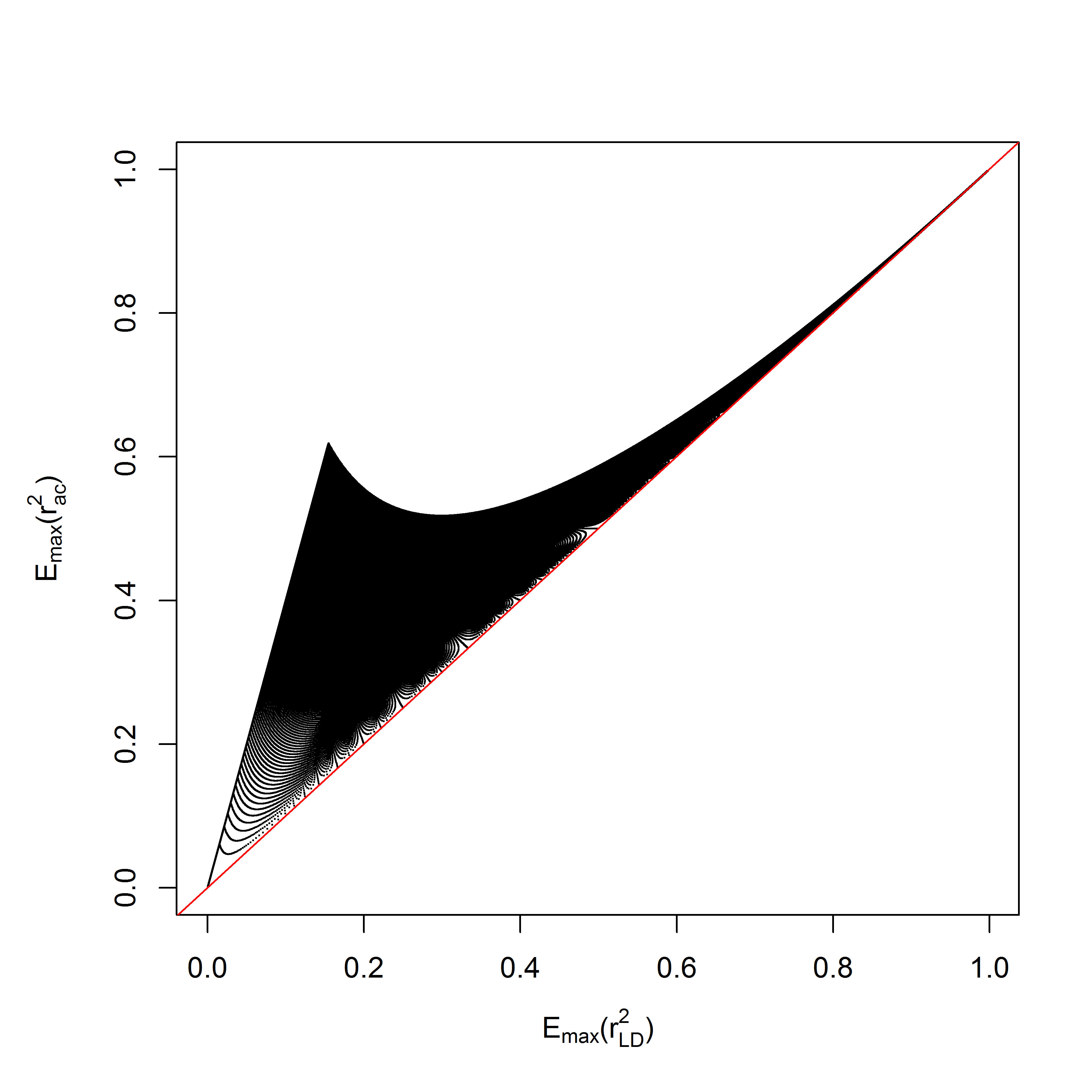


Figure S1 – Comparison of values of $E_{max}\left( r_{LD}^{2} \right)$ and $E_{max}\left( r_{ac}^{2} \right)$ for values of $E_{max}\left( r_{LD}^{2} \right)$ ranging from 0 and 1. Pairs of $r^{2}$ values are indicated by black dots. The red line indicates $r_{ac}^{2}=r_{LD}^{2}$ as a reference.


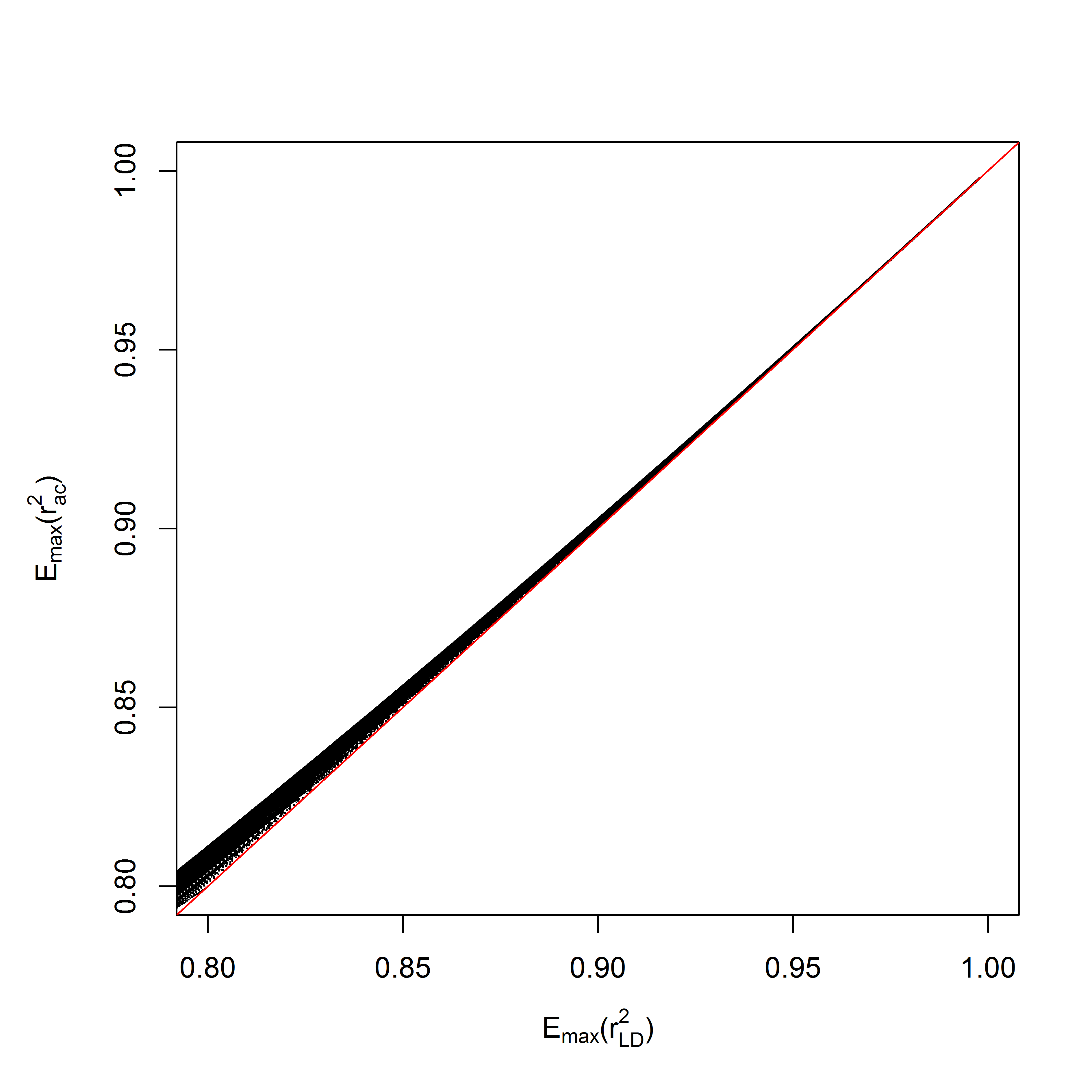


Figure S2 – Comparison of values of $E_{max}\left( r_{LD}^{2} \right)$ and $E_{max}\left( r_{ac}^{2} \right)$ for values of $E_{max}\left( r_{LD}^{2} \right)$ ranging from 0.8 and 1. Pairs of $r^{2}$ values are indicated by black dots. The red line indicates $r_{ac}^{2}=r_{LD}^{2}$ as a reference.


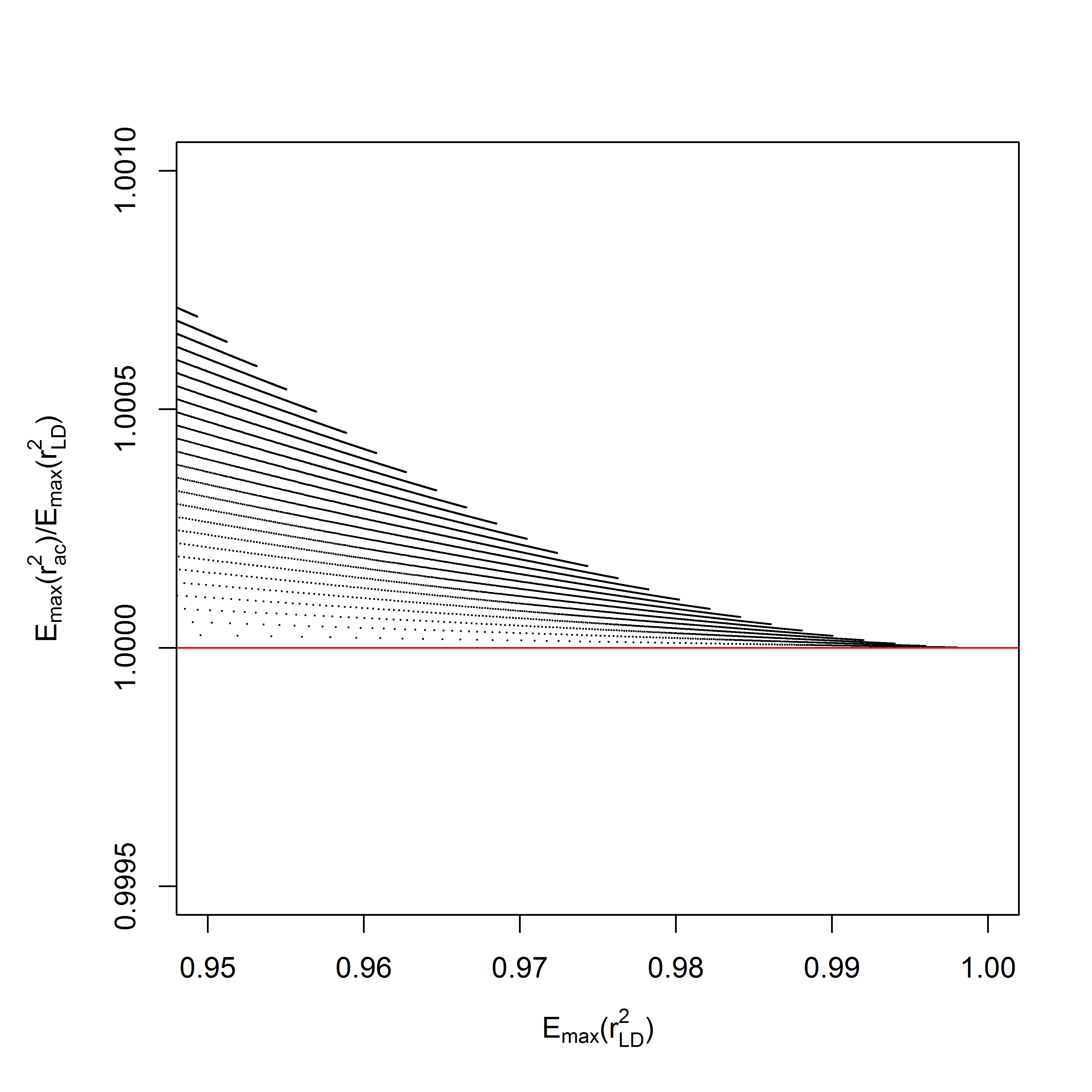


Figure S3 – The ratio $E_{max}\left( r_{LD}^{2} \right)/E_{max}\left( r_{LD}^{2} \right)$ for values of $E_{max}\left( r_{LD}^{2} \right)$ ranging from 0.95 to 1. Pairs of $r^{2}$ values are indicated by black dots. The red line indicates $r_{ac}^{2}=r_{LD}^{2}$ as a reference.
